# Long-term ambient air pollution exposure and prospective change in sedentary behaviour and physical activity in individuals at risk of type 2 diabetes in the UK

**DOI:** 10.1093/pubmed/fdad263

**Published:** 2023-12-16

**Authors:** Jonathan Goldney, Joseph Henson, Charlotte L Edwardson, Kamlesh Khunti, Melanie J Davies, Thomas Yates

**Affiliations:** Diabetes Research Centre, College of Life Sciences, University of Leicester, Gwendolen Rd, Leicester LE5 4PW, UK; Diabetes Research Centre, College of Life Sciences, University of Leicester, Gwendolen Rd, Leicester LE5 4PW, UK; NIHR Leicester Biomedical Research Centre, University Hospitals of Leicester NHS Trust and University of Leicester, Gwendolen Rd, Leicester LE5 4PW, UK; Diabetes Research Centre, College of Life Sciences, University of Leicester, Gwendolen Rd, Leicester LE5 4PW, UK; NIHR Leicester Biomedical Research Centre, University Hospitals of Leicester NHS Trust and University of Leicester, Gwendolen Rd, Leicester LE5 4PW, UK; Diabetes Research Centre, College of Life Sciences, University of Leicester, Gwendolen Rd, Leicester LE5 4PW, UK; NIHR Leicester Biomedical Research Centre, University Hospitals of Leicester NHS Trust and University of Leicester, Gwendolen Rd, Leicester LE5 4PW, UK; Leicester Real World Evidence Unit, Leicester Diabetes Centre, University of Leicester, Gwendolen Rd, Leicester LE5 4PW, UK; Diabetes Research Centre, College of Life Sciences, University of Leicester, Gwendolen Rd, Leicester LE5 4PW, UK; NIHR Leicester Biomedical Research Centre, University Hospitals of Leicester NHS Trust and University of Leicester, Gwendolen Rd, Leicester LE5 4PW, UK; Diabetes Research Centre, College of Life Sciences, University of Leicester, Gwendolen Rd, Leicester LE5 4PW, UK; NIHR Leicester Biomedical Research Centre, University Hospitals of Leicester NHS Trust and University of Leicester, Gwendolen Rd, Leicester LE5 4PW, UK

**Keywords:** air pollution, chronic disease, physical activity

## Abstract

**Background:**

Air pollution may be a risk factor for physical inactivity and sedentary behaviour (SED) through discouraging active lifestyles, impairing fitness and contributing to chronic diseases with potentially important consequences for population health.

**Methods:**

Using generalized estimating equations, we examined the associations between long-term particulate matter with diameter ≤2.5 μm (PM2.5), ≤10 μm (PM10) and nitrogen dioxide (NO_2_) and annual change in accelerometer-measured SED, moderate-to-vigorous physical activity (MVPA) and steps in adults at risk of type 2 diabetes within the Walking Away from Type 2 Diabetes trial. We adjusted for important confounders including social deprivation and measures of the built environment.

**Results:**

From 808 participants, 644 had complete data (1605 observations; 64.7% men; mean age 63.86 years). PM2.5, NO_2_ and PM10 were not associated with change in MVPA/steps but were associated with change in SED, with a 1 ugm^−3^ increase associated with 6.38 (95% confidence interval: 0.77, 12.00), 1.52 (0.49, 2.54) and 4.48 (0.63, 8.34) adjusted annual change in daily minutes, respectively.

**Conclusions:**

Long-term PM2.5, NO_2_ and PM10 exposures were associated with an annual increase in SED: ~11–22 min/day per year across the sample range of exposure (three standard deviations). Future research should investigate whether interventions to reduce pollution may influence SED.

## Introduction

Physical activity (PA; any bodily movement produced by skeletal muscles that requires energy expenditure^1^) and sedentary behaviour (SED; any waking behaviour done whilst lying, reclining, sitting or standing, with no ambulation, irrespective of energy expenditure^2^) are independently associated with numerous diseases and all-cause mortality.^3–6^ Physical inactivity is prevalent with self-reported data suggesting that worldwide, 27.5% of adults were not meeting global recommendations for PA.^7^

Similarly, ambient air pollution is associated with cardiometabolic and respiratory diseases,^8–10^ and is extremely prevalent: in 2019 the World Health Organization estimated that 99% of the global population breathe air containing high levels of pollutants.^11^ Both household air pollution (polluting fuels used for cooking or heating) and ambient (outdoor) air pollution pose risks to health, together associated with 6.7 million premature deaths a year globally.^8^^,^^11^ Particulate matter with diameter ≤2.5 μm (PM2.5), ≤10 μm (PM10), and nitrogen dioxide (NO_2_) are the most measured air pollutants in health research.^12–14^ Whilst studies may focus on a specific pollutant exposure, exposures to different pollutants are highly colinear, meaning that the individual effect of each pollutant is difficult to discern in observational research.^10^

The harmful associations of physical inactivity, SED and air pollution, may not be independent. There are several hypotheses regarding a multi-faceted bidirectional relationship.^15^ For example, active transport may result in fewer vehicle emissions and may resultingly lower local exposure to air pollution.^15^ Conversely, PA may increase the harmful effects of air pollution,^16^ due to an increased exposure of respiratory mucosa to air pollution through increased minute volume, resulting in an increase in systemic inflammation.^17^ This may explain some evidence suggesting that the benefits of PA are attenuated at high concentrations of ambient air pollution.^18^^,^^19^

Additionally, air pollution may be a risk factor for physical inactivity and SED, through discouraging active lifestyles, impairing cardiorespiratory fitness and contributing to chronic diseases^15^^,^^20^; air pollution may increase physical inactivity/SED and may further increase chronic disease risk in a potential positive feedback loop.^8–10^^,^^21^ The relationships between air pollution and PA/SED are crucial to ascertain, particularly in view of climate change. For instance, more extreme temperatures and more frequent natural disasters, may decrease PA further, over and above a direct association between increased air pollutant concentration (frequently greenhouse gases) and decreased PA.^22^

Most evidence exploring the association between PA/SED and air pollution is limited by cross-sectional design and use of subjectively measured PA/SED.^15^^,^^20^ Whilst cross-sectional studies are useful at detecting associations, they are less useful in describing how interventions to modify exposures may influence outcomes, should they be causally associated; prospective data allows for inference on how an ongoing exposure (e.g. higher concentrations of air pollution) may have an ongoing effect on an outcome (e.g. change in PA/SED). Furthermore, whilst there has been an increase in literature utilizing accelerometer-measured PA/SED,^23–25^ to the authors’ knowledge, there are no studies utilizing both accelerometer measures and a prospective design to examining the relationship between air pollution and PA/SED, particularly in those at high risk of metabolic diseases.

To address this gap, we investigated whether concentrations of PM2.5, NO_2_ and PM10 were associated with year-on-year change in accelerometer-measured moderate-to-vigorous PA (MVPA)/SED/steps in individuals at risk of type 2 diabetes (T2D); with the hypothesis that pollutant concentrations will be negatively associated with change in PA (MVPA and steps), and positively associated with change in SED.

## Materials and methods

### Cohort definition

This study utilized data from the ‘Walking Away from Type 2 Diabetes’ cluster-randomized control trial (Clinical trial: *NCT00941954*) which randomized 10 general practices throughout Leicestershire, UK, between 2009 and 2010, to either the ‘Walking Away from Type 2 Diabetes’ intervention (a low-cost 3-h group-based education programme, pedometers and annual refresher sessions) or standard care. Participants were eligible between the ages of 18–74 years that were at high risk for T2D, above the 90th percentile of the Leicester Practice Risk Score.^26^

Measurements were taken at baseline, 12, 24 and 36 months. Methods have been described extensively elsewhere.^27^ The study was conducted according to the guidelines laid down in the Declaration of Helsinki and all participants provided written informed consent. The Nottingham Research Ethics Committee 2 and the Leicestershire, Northamptonshire and Rutland Comprehensive Local Research network, gave full ethical and governance approval in April 2009 (09/H0408/32).

The intervention led to a modest increase in steps/day at 12 months however it did not lead to a sustained change over a 3-year period in MVPA, SED or steps^28^; therefore, control and intervention groups were combined into a single cohort for the purposes of this analysis.

### Outcome

Changes in daily minutes of MVPA, SED and steps across each 12-month period within the study duration were the primary outcomes in this analysis. Each participant therefore had up to three observations: change between 0–12, 12–24 and 24–36 months.

Participants wore a waist-worn accelerometer (GT3X; ActiGraph, Pensacola, FL, USA) on their right anterior axillary line for seven consecutive days during waking hours. At least four valid days of data were required (minimum ≥10 h/day) for inclusion. Data were recorded in 15 s epochs then re-integrated into 60 s epochs for processing using a commercially available data analysis tool that converts the ActiGraph counts into more usable metrics (KineSoft version 3.3.76, KineSoft, Loughborough, UK; www.kinesoft.org). Sedentary time was defined by the intensity thresholds of <100 counts/min and MVPA ≥1952 counts/min.^27^^,^^29^

### Ambient air pollution

We investigated exposure to PM2.5, NO_2_ and PM10 as components of ambient air pollution. The 3-year average concentrations from the year the participant entered the study and the preceding 2 years (to represent meaningful exposure) were calculated. These estimates were sourced from the Department for Environment Food and Rural Affairs (DEFRA) Pollution Climate Mapping model. The models are run by Ricardo Energy & Environment (Oxfordshire, UK) on behalf of DEFRA. DEFRA publishes annual pollutant concentrations in 1 × 1 km grids. The concentrations published for the grid containing the participant’s postal code were used.

For the purposes of sensitivity analysis, the average pollutant concentration was estimated during each observation period using a weighted average of the published data for the 2 years for which the observation occurred within. The weighting was dependent on the proportion of time the 12-month observation fell within each year.

### Confounding factors

Sex (men/women), ethnicity (white/south Asian/other), age, smoking status (current smoker/non-smoker), past medical history of cardiovascular and respiratory disease (yes/no) and occupational status (full time work >30 h per week/ part time work/ keeping house/ retired/ waiting to start a new job already obtained/ unemployed and looking for work/ out of work as temporarily sick/ permanently sick or disabled) were collected via an interview-administered questionnaire.^27^ Ethnicity was collected as census categories and grouped as follows: white (white British, white Irish, other white background), south Asian (Indian, Pakistani, Bangladeshi, other Asian background) and other.

Body weight (Tanita TBE 611, Tanita, West Drayton, UK) and height were measured by trained staff to the nearest 0.1 kg and 0.5 cm, respectively, and used to calculate body mass index (BMI).

Social deprivation was estimated using the English Indices of Deprivation 2010 which provides a relative measure of deprivation at small area level across England and comprises seven domains: Income Deprivation, Employment Deprivation, Health Deprivation and Disability, Education Skills and Training Deprivation, Barriers to Housing and Services, Living Environment Deprivation, and Crime.^30^ Social deprivation is associated with both pollutant concentration and PA/SED and may therefore be a confounder.^31^^,^^32^

Data were also collected for neighbourhood greenspace. The UK Ordnance Survey Code-point database was used to obtain the average coordinates of a postcode, containing ~15 addresses.^33^ ArcGIS 10.1 (ESRI, California, USA), a geographic information system (GIS), was used to generate a circle centred around a participant’s postal code with a radius of 800 m, with data generated in 2015.^34^ An 800 m radius was chosen as this has been used as a typical definition for a neighbourhood, approximating to a 10-min walk.^35^ Greenspace, as a percentage, was then computed through imposing this 800 m radius onto the mapped estimates of greenspace published by the Centre for Ecology and Hydrology Land Cover Map of the UK in 2011 within the GIS.^36^ Greenspace may also represent an important confounder: associated positively with PA, negatively with SED and found to mediate the relationship between air pollution and disease.^37–45^

Measures of the built environment were computed using the UK Ordnance Survey Code-point database within the GIS. These included: Road density and footpath density (the length of roads/footpaths (m) within a radius of 800 m from a participant’s postal code), junctions and cul-de-sacs (the number of road junctions/cul-de-sacs within a radius of 800 m from a participant’s postal code), and connected intersections (the ratio of junctions leading to a further junction and junctions leading to a cul-de-sac within 800 m from a participant’s postal code). Measures of the built environment have been shown to be associated with PA/SED and may therefore also be confounders.^46–49^

### Statistical analysis

Baseline characteristics were summarized, including the number of participants exposed to an annual average of PM2.5/NO_2_/PM10 that this harmful to health, defined by the UK/European thresholds.^50^

After ensuring a linear relationship between exposures and outcomes, generalized estimating equations were used to predict the change in MVPA/SED/steps from pollutant concentrations. An exchange correlation matrix was fitted across three levels as has been reported previously^51^: change between 0–12, 12–24 and 24–36 months.

Two models were used. Model 1 adjusted for baseline demographic factors: age, ethnicity, sex, smoking status, past medical history of cardiovascular disease, past medical history of respiratory disease; as well as BMI, season and MVPA/SED/steps at the start of index observation period; treatment group (intervention/control) and change in wear time between accelerometery measures. Model 2 further adjusted for socioeconomic confounders (social deprivation and occupational status), greenspace and measures of the built environment (road density, footpath density, junctions, cul-de-sacs and connected intersections). NO_2_, PM2.5 and PM10 exposures were analyzed in separate models due to collinearity (Supplementary Table 1).

**Fig. 1:**
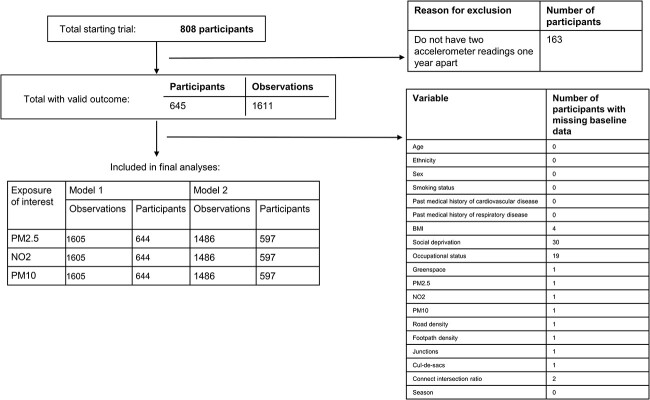
Cohort definition. BMI: Body Mass Index; PM2.5: Particulate matter ≤2.5 μm; NO_2_: Nitrogen dioxide; PM10: particulate matter ≤10.0 μm; SED: Time spent in sedentary behaviour; MVPA: Time spent in moderate-to-vigorous physical activity.

For visual purposes and to compare differences in outcomes across exposures, values for pollutants and changes in MVPA/SED/steps were converted to a z-score by subtracting the mean from the raw score and dividing by the standard deviation (SD). Models were repeated to give change in outcome per 1SD increase in exposure to air pollutants. A sensitivity analysis was also undertaken, to investigate whether average pollutant exposure during the observation may have a different association with change in MVPA/SED/steps, as compared to pollutant exposure in the preceding 3-years using the same models. A complete case analysis was undertaken as a final sensitivity analysis. For all analyses the level of significance was set at *P* < 0.05. All analyses were undertaken in IBM SPSS (version 26) and presented visually using GraphPad Prism 9.4.1.

## Results

From 10 general practices, 808 participants were recruited. A total of 163 participants did not have sufficient accelerometer data to be included, others had an observation(s) missing for BMI and/or air pollutant data which were further excluded (although observations with complete data remained; Fig. 1). There were 644 participants with 1605 observations (accelerometer readings 1 year apart) included in model 1, and 597 participants with 1486 observations in model 2 following further missing socioeconomic and built environment data (Fig. 1).

Table 1 shows the characteristics of included participants, with excluded participant characteristics presented in Supplementary Table 2. In the included participants, the mean age (SD) was 64.08 (7.37) years, 417 (64.8%) were men and 227 (35.2%) were women. Most of the participants were white (573, 89.0%), followed by south Asian (42, 6.5%) and other (29, 4.5%). Excluded participants were slightly younger (mean of 61.34 years), had slightly fewer men (59.1%), slightly more women (40.9%), more people of south Asian ethnicity (10.4%) and less other ethnicity (1.8%). They additionally had higher social deprivation and had a higher BMI. The 3-year average pollutant concentrations that participants were exposed to were 11.80μgm^−3^ (SD: 0.66), 21.28μgm^−3^ (4.74) and 16.15μgm^−3^ (0.84) for PM2.5, NO_2_ and PM10, respectively. None of the participants were exposed to average PM2.5, NO_2_ or PM10 participant concentrations above concentrations harmful for health.

**Table 1:** Participant characteristics included in the analysis

Characteristic		Participants
Number		644
Age (years)		64.08 (7.37)
Sex	Men	417 (64.8%)
	Women	227 (35.2%)
Ethnicity	White	573 (89.0%)
	South Asian	42 (6.5%)
	Other	29 (4.5%)
Smoking status	Smoker	49 (7.6%)
	Non-smoker	595 (92.4%)
Past medical history of cardiovascular disease		208 (32.3%)
Past medical history of respiratory disease		14 (2.2%)
Body Mass Index (kg/m^2^)		31.64 (5.22)
Occupational status	Full time work (>30 h per week)	151 (24.2%)
	Part time work (<30 h per week)	67 (10.7%)
	Keeping house	18 (2.9%)
	Retired	360 (57.6%)
	Waiting to start a new job already obtained	1 (0.2%)
	Unemployed and looking for work	10 (1.6%)
	Out of work as temporarily sick	6 (1.0%)
	Permanently sick or disabled	12 (1.9%)
	Missing	151 (24.2%)
Social deprivation (Townsend deprivation index)		12.86 (4.84, 52.90)
Greenspace (%)		10.20 (3.92)
Road density (km)		0.48 (0.00, 2.34)
Footpath density (km)		5.40 (1.11)
Junctions (*n*)		2.26 (0.98)
Cul-de-sacs (*n*)		0.72 (0.13)
Connected intersection ratio		12.86 (4.84, 52.90)
Average PM2.5 concentration in 3-years prior to observation (μgm^−3^)		11.80 (0.66)
Average NO_2_ concentration in 3-years prior to observation (μgm^−3^)		21.28 (4.74)
Average PM10 concentration in 3-years prior to observation (μgm^−3^)		16.15 (0.84)
Estimated PM2.5 concentration over observation periods (μgm^−3^)		12.07 (0.85)
Estimated NO_2_ concentration over observation periods (μgm^−3^)		20.80 (4.72)
Estimated PM10 concentration over observation periods (μgm^−3^)		16.98 (1.30)
Participants receiving intervention		327 (50.8%)
MVPA at start of observations (minutes/day)		20.00 (1.62, 73.20)
SED at start of observations (minutes/day)		548.88 (91.26)
Steps at start of observation (steps/day)		−1.81 (16.89)
Change in MVPA (minutes/day)		7.61 (70.73)
Change in SED (minutes/day)		−219.22 (1942.25)
Change in steps (steps/day)		6610.17 (3113.80)

Data presented as:

Categorical: number (percentage of total).

Normally distributed variable: mean (SD).

Non-normal distributed variable: median (5th, 95th centile).

PM2.5: Particulate matter ≤2.5 μm; NO_2_: Nitrogen dioxide; PM10: particulate matter ≤10.0 μm; SED: Time spent in sedentary behaviour; MVPA: Time spent in moderate-to-vigorous physical activity.

The associations between environmental exposures and change in MVPA, SED and steps across increasing levels of adjustment are shown in Table 2. Air pollutants were not associated with change in MVPA or steps but were all associated with change in SED in both models, with results for model 2 showing a 6.38 (95% confidence interval [CI]: 0.77, 12.00), 1.52 (0.49, 2.54) and 4.48 (0.63, 8.34) annual change in daily minutes of SED per each 1 ugm^−3^ increase in PM2.5, NO_2_ and PM10, respectively.

**Table 2:** Association between average pollutant concentrations in the 3-years before observation and change in annual change in daily minutes of MVPA/ SED and steps

	Model 1						Model 2					
	Change in MVPA (min/day)		Change in SED (min/day)		Change in steps (steps/day)		Change in MVPA (min/day)		Change in SED (min/day)		Change in Steps (steps/day)	
	β (95% CI)	*P* =	β (95% CI)	*P* =	β (95% CI)	*P* =	β (95% CI)	*P* =	β (95% CI)	*P* =	β (95% CI)	*P* =
3-year average PM2.5 (μgm^−3^)	−0.13 (−1.04, 0.77)	0.770	**5.27 (0.92, 9.62)**	**0.017** *	−4.55 (−109.14, 100.05)	0.932	−0.49 (−1.68, 0.71)	0.425	**6.38 (0.77, 12.00)**	**0.026** *	−22.61 (−163.88, 118.67)	0.754
3-year average NO_2_ (μgm^−3^)	−0.02 (−0.15, 0.11)	0.721	**0.85 (0.24, 1.46)**	**0.006** *	−3.99 (−17.88, 9.91)	0.574	−0.15 (−0.36, 0.05)	0.136	**1.52 (0.49, 2.54)**	**0.004** *	−17.60 (−41.02, 5.82)	0.141
3-year average PM10 (μgm^−3^)	−0.26 (−0.96, 0.43)	0.460	**4.18 (0.79, 7.58)**	**0.016** *	−16.74 (−98.07, 64.58)	0.687	−0.41 (−1.23, 0.40)	0.323	**4.48 (0.63, 8.34)**	**0.023** *	−20.71 (−115.55, 74.14)	0.669

^*^Represents significant values (*P* < 0.05).

β represents the change in MVPA/SED/steps per 1 μgm^−3^ increasing in pollutant concentration.

Model 1: Standard adjustments (Age, ethnicity, sex, smoking status, past medical history of cardiovascular disease, past medical history of respiratory disease, treatment group, change in wear time between accelerometery measures, SED/MVPA/steps at start of observation, body mass index, season of accelerometer measurements).

Model 2: Standard adjustments, social deprivation, greenspace, measures of built environment (road density, footpath density, junctions, cul-de-sacs, connected intersections), occupational status.

MVPA: Time spent in moderate-to-vigorous physical activity; SED: Time spent in sedentary behaviour; CI: Confidence interval; PM2.5: Particulate matter ≤2.5 μm; NO_2_: Nitrogen dioxide; PM10: particulate matter ≤10.0 μm.

**Fig. 2:**
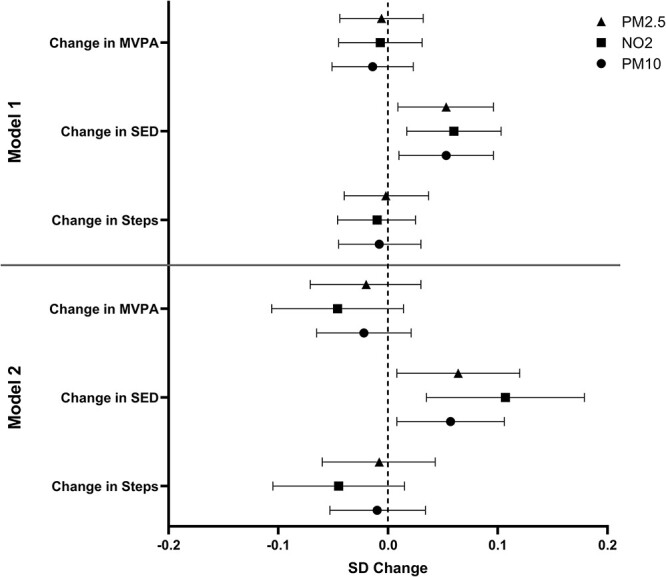
SD change in daily MVPA and SED per one standard deviation increase in pollutant concentration. Bars represent 95% confidence interval. Model 1: Standard adjustments (Age, ethnicity, sex, smoking status, past medical history of cardiovascular disease, past medical history of respiratory disease, treatment group, change in wear time between accelerometery measures, SED/MVPA/steps/LPA at start of observation, body mass index, season of accelerometer measurements). Model 2: Standard adjustments + social deprivation + greenspace + measures of built environment (road density, footpath density, junctions, cul-de-sacs, connected intersections) + occupation. MVPA: Time spent in moderate-to-vigorous physical activity; SED: Time spent in sedentary behaviour; PM2.5: Particulate matter ≤2.5 μm; NO_2_: Nitrogen dioxide; PM10: particulate matter ≤10.0 μm; SD: Standard deviation.

Similar findings were observed after exposures and outcomes were converted to z-scores (Supplementary Table 3**;**  Fig. 2): for PM2.5, NO_2_ and PM10 in model 2, a 0.06 (95% CI, 0.01, 0.12), 0.11 (0.03, 0.18), 0.06 (0.01, 0.11) SD annual increase in SED per 1SD increase in pollutant concentration.

In the sensitivity analysis, using the average pollutant concentration during the observation (rather than the 3-year average preceding), the results were unchanged in terms of significance and effect size from the main analysis (Supplementary Table 4). Similarly, results from the sensitivity analyses involving complete case analysis were not substantially different (Supplementary Table 5).

## Discussion

### Main findings of this study

We show that PM2.5, NO_2_ and PM10 were associated with annual change in daily minutes of SED but not with MVPA or steps, independent of multiple confounders, in participants at high risk of T2D. We observed this association regardless of how concentrations of pollutants were measured, including as a 3-year average (year of start of observation with the two preceding years), or as the average pollutant concentration during the 12-month observation period.

An increase of 1 μgm^−3^ in the average concentration of atmospheric NO_2_ was associated with 1.52 (95% CI: 0.49, 2.54) min/day annual increase in SED in the most adjusted model. Across the cohort, our findings suggest that high versus low exposure (three SD difference) to NO_2_ (1SD = 4.74μgm^−3^) could be associated with 22 min/day of increased SED per year. Similarly, high versus low exposure to PM2.5 (3SD = 1.98μgm^−3^) and PM10 (2.52μgm^−3^) in our cohort could be associated with 13 and 11 min/day respectively of increased SED annually. In less-active adults, replacing 1 h/day of SED with light PA has been associated with an 18% lower all cause-mortality.^52^ Our findings could have significant implications for prevention if causal, as interventions to reduce ambient air pollution concentration are likely to modify the exposure of numerous individuals, particularly in urban locations, and so a modest positive effect on each individual’s SED may surmount to a large public health effect.

### What is already known on this topic

Few studies exist investigating the relationship between air pollution and SED, particularly prospective designs, however they are largely consistent with our findings. One prospective study of 9700 students in Beijing found that PM2.5, PM10, NO_2_ concentrations were positively associated with self-reported SED, albeit to a far greater magnitude than the present analysis (6.24–7.06 h/week per 1SD increase) possibly reflecting a higher average pollutant concentration.^53^ Similar findings with PM2.5 and accelerometer measured SED were found in a cross-sectional study of 340 students in Beijing.^24^

Conversely, our findings of a lack of association between pollutants and PA is generally not consistent with the literature. One prospective study in 73 participants with chronic obstructive pulmonary disease (COPD) using pedometer data found that ozone (O_3_) was associated with a lower number of steps/day over a week, and PM10 with lower steps/day during the weekdays but not over the full week.^54^ Similarly, another cross-sectional study in 418 participants with COPD found that O_3_ was negatively associated with self-reported PA.^55^ Similar findings have been found within two large UK Biobank cross-sectional studies utilizing accelerometer measures: in 65 967 participants, concentration of nitrogen oxides ≥26 μgm^−3^ was associated with lower levels of PA after adjusting for greenness and area-level deprivation^23^; and in 84 052 participants, PM2.5 and PM10 were associated with lower MVPA adjusted for age, sex and ethnicity.^56^ Similar negative associations were found in smaller cross-sectional studies with accelerometer-measured PA from samples in Seattle, USA (*n* = 288; NO_2_) and students in Beijing, China (*n* = 340; air quality index and PM2.5).^24^^,^^25^ As mentioned, in Beijing, the concentration of PM2.5 was far higher than in the present analysis, with a mean concentration of 68.0 μgm^−3^ compared with 11.80 μgm^−3^, and larger sample variation, potentially giving greater statistical power to identify differences. Furthermore, air pollution may only become detrimental to PA above a certain threshold that was not met within our study.

Other reasons for the discrepancy between our findings in relation to PA and previous literature may include the relative low levels of baseline MVPA/steps, with less opportunity to reduce discretionary levels of MVPA/steps and larger opportunity to increase SED. Purposeful PA may also be undertaken away from a participant’s postal area (where air pollution is measured), meaning measured air pollution may capture actual exposure differently for different populations. Similarly, household air pollution was not accounted for. The differential association of air pollution with SED and MVPA/steps requires further investigation in other clinical populations.

### What this study adds

This study builds on emerging evidence that ambient air pollution is positively associated with SED, utilizing a prospective design, large cohort and accelerometer-measured SED adjusted for multiple potential confounders. Our findings of a lack of association between air pollution and MVPA/steps are discrepant with much of the literature and therefore highlight that future research should investigate if and how this association may vary in different populations.

Our findings also raise the question of whether low SED could be mediator of the association between air pollution and cardiometabolic risk. However, one previous study found only a minor attenuation of the increased risk of cardiometabolic disease when lifestyle factors were adjusted for (although only self-reported PA was used, not SED).^57^

### Limitations of this study

Causality cannot be established due to the observational nature of this study and so should be used for hypothesis generation only, due to residual or unmeasured confounding, such as community-level use of automated transport or noise pollution.^58^ Despite this, we adjusted for numerous possible confounding factors, including social deprivation and measures of the built environment.

The area-level measures of social deprivation and pollution may also incorrectly categorize individual participants and introduce ecological bias. The measures of pollution also do not account for pollutant exposure away from the home and do not capture household air pollution. Similarly, we do not know where participants undertook PA/SED, and whether pollutants had differing effects on PA/SED dependent on indoor/outdoor location. Furthermore, we were unable to adjust our models by other pollutants due to collinearity, making assessment of the relative risk contribution of each difficult. We were unable to determine the shorter-term effects of pollutant concentration on physical activity as accelerometers were only worn on an annual basis.

The generalisability is also limited by loss of data, and having focused on a particular geographical area within a temperate climatic region, with average pollution levels well below consensus targets for health, in those with a high risk of chronic disease, however both urban and rural populations were included.

## Conclusions

Our findings suggest that measures of air pollution, PM2.5, NO_2_ and PM10, are associated with an annual increase in accelerometer-measured SED but not with change in MVPA or steps. Our findings warrant further investigation of this relationship in other populations and whether interventions to reduce pollutant concentrations may influence SED.

## Supplementary Material

Supplementary_Tables2_clean_fdad263

STROBE-checklist_fdad263

## Data Availability

The data underlying this article will be shared on reasonable request to the corresponding author.

## Notes

**Jonathan Goldney,** NIHR Academic Clinical Fellow

**Joseph Henson,** Research Associate

**Charlotte L. Edwardson,** Professor in Sedentary Behaviour and Health

**Kamlesh Khunti,** Professor of Primary Care Diabetes and Vascular Medicine

**Melanie J. Davies,** Professor of Diabetes Medicine

**Thomas Yates,** Professor of Physical Activity, Sedentary Behaviour and Health
