# Supplementary material for: Long-term ambient air pollution exposure and prospective change in sedentary behaviour and physical activity in individuals at risk of type 2 diabetes in the UK
Source: J Public Health (Oxf). 2023 Dec 16;46(1):e32–42. doi: 10.1093/pubmed/fdad263 (PMC10901272; doi:10.1093/pubmed/fdad263)
Supplement: Supplementary_Tables2_clean_fdad263 [file supplementary_tables2_clean_fdad263.docx]

Supplementary Tables

**Ambient air pollution exposure and prospective change in sedentary behaviour and moderate-to-vigorous physical activity in individuals at risk of type 2 diabetes**

Jonathan Goldney, Joseph Henson, Charlotte L. Edwardson, Kamlesh Khunti, Melanie J. Davies, Thomas Yates

Contents

[Supplementary Table 1: Correlation matrix between air pollutant concentrations 2](#_Toc149054361)

[Supplementary Table 2: Participant characteristics included in analysis compared with total trial population 3](#_Toc149054362)

[Supplementary Table 3: Annual change in standard deviations of MVPA/SED/steps per one standard deviation increase in 3-yr average pollutant concentrations 5](#_Toc149054363)

[Supplementary Table 4: Association between average pollutant concentrations during observation and change in annual change in daily minutes of MVPA/SED/steps 6](#_Toc149054364)

[Supplementary Table 5: Complete case analysis for the associations of exposure to air pollution and annual change in daily minutes of MVPA/SED/steps. 7](#_Toc149054365)

# Supplementary Table 1: Correlation matrix between air pollutant concentrations

|  | **PM2.5** | **NO2** | **PM10** |
| --- | --- | --- | --- |
| **PM2.5** | 1 | 0.823 | 0.952 |
| **NO2** | 0.823 | 1 | 0.672 |
| **PM10** | 0.952 | 0.672 | 1 |

Values represent Pearson Correlation Coefficient

**PM2.5:** Particulate matter ≤ 2.5μm; **NO2:** Nitrogen dioxide; **PM10:** particulate matter ≤ 10.0μm

# Supplementary Table 2: Participant characteristics included in analysis compared with total trial population

| **Characteristic** | | | **Included participants** | **Excluded participants** | **Total** |
| --- | --- | --- | --- | --- | --- |
| Number | | | 644 | 164 | 808 |
| Age (years) | | | 64.08 (7.37) | 61.34 (9.24) | 63.15 (8.15) |
| Sex | | Men | 417 (64.8%) | 97 (59.1%) | 514 (63.6%) |
|  |  | Women | 227 (35.2%) | 67 (40.9%) | 294 (36.4%) |
| Ethnicity | | White | 573 (89.0%) | 144 (87.8%) | 717 (88.7%) |
|  |  | South Asian | 42 (6.5%) | 17 (10.4%) | 59 (7.3%) |
|  |  | Other | 29 (4.5%) | 3 (1.8%) | 32 (4.0%) |
| Smoking status | | Smoker | 49 (7.6%) | 29 (17.7%) | 78 (9.7%) |
|  |  | Non-smoker | 595 (92.4%) | 135 (82.3%) | 730 (90.3%) |
| Past medical history of cardiovascular disease | | | 208 (32.3%) | 49 (29.9%) | 257 (31.8%) |
| Past medical history of respiratory disease | | | 14 (2.2%) | 2 (1.2%) | 16 (2.0%) |
| Body Mass Index (kg/m²) | | | 31.64 (5.22) | 33.32 (6.11) | 32.06 (5.51) |
| Occupational status | Full time work (>30 hours per week) | | 151 (24.2%) | 38 (24.7%) | 189 (24.3%) |
|  | Part time work (<30 hours per week) | | 67 (10.7%) | 20 (13.0%) | 87 (11.2%) |
|  | Keeping house | | 18 (2.9%) | 4 (2.6%) | 22 (2.8%) |
|  | Retired | | 360 (57.6%) | 73 (47.4%) | 433 (55.6%) |
|  | Waiting to start a new job already obtained | | 1 (0.2%) | 0 (0.0%) | 1 (0.1%) |
|  | Unemployed and looking for work | | 10 (1.6%) | 2 (1.3%) | 12 (1.5%) |
|  | Out of work as temporarily sick | | 6 (1.0%) | 4 (2.6%) | 10 (1.3%) |
|  | Permanently sick or disabled | | 12 (1.9%) | 13 (8.4%) | 25 (3.2%) |
|  | Missing | | 151 (24.2%) | 38 (24.7%) | 189 (24.3%) |
| Social deprivation (Townsend deprivation index) | | | 12.86 (4.84, 52.90) | 17.34 (5.52, 64.60) | 13.95 (5.11, 53.50) |
| Greenspace (%) | | | 42.41 (26.17) | 40.82 (27.98) | 41.88 (26.80) |
| Road density (km) | | | 10.20 (3.92) | 10.53 (4.32) | 10.31 (4.06) |
| Footpath density (km) | | | 0.48 (0.00, 2.34) | 0.49 (0.00, 2.36) | 0.48 (0.00, 2.34) |
| Junctions (*n*) | | | 5.40 (1.11) | 5.44 (1.26) | 5.41 (1.16) |
| Cul-de-sacs (*n*) | | | 2.26 (0.98) | 2.21 (1.09) | 2.24 (1.02) |
| Connected intersection ratio | | | 0.72 (0.13) | 0.72 (0.14) | 0.72 (0.13) |
| Average PM2.5 concentration in 3-years prior to observation (µgm^-3^) | | | 11.80 (0.66) | 11.91 (0.80) | 11.83 (0.71) |
| Average NO2 concentration in 3-years prior to observation (µgm^-3^) | | | 21.28 (4.74) | 21.8 (5.41) | 21.45 (4.98) |
| Average PM10 concentration in 3-years prior to observation (µgm^-3^) | | | 16.15 (0.84) | 16.28 (1.00) | 16.2 (0.90) |
| Estimated PM2.5 concentration over observation periods (µgm^-3^) | | | 12.07 (0.85) | 12.14 (0.97) | 12.09 (0.90) |
| Estimated NO2 concentration over observation periods (µgm^-3^) | | | 20.80 (4.72) | 21.25 (5.36) | 20.95 (4.95) |
| Estimated PM10 concentration over observation periods (µgm^-3^) | | | 16.98 (1.30) | 16.99 (1.39) | 16.98 (1.33) |
| Participants receiving intervention | | | 327 (50.8%) | 96 (58.5%) | 423 (52.4%) |

Data presented as:
- Categorical: number (percentage of total)
- Normally distributed variable: mean (Standard deviation)
- Non-normal distribution: median (5^th^, 95^th^ centile)

**PM2.5:** Particulate matter ≤ 2.5μm; **NO2:** Nitrogen dioxide; **PM10:** particulate matter ≤ 10.0μm

Supplementary Table 3: Annual change in standard deviations of MVPA/SED/steps per one standard deviation increase in 3-yr average pollutant concentrations

|  | **Model 1** | | | | | | **Model 2** | | | | | |
| --- | --- | --- | --- | --- | --- | --- | --- | --- | --- | --- | --- | --- |
|  | Change in MVPA (SD) | | Change in SED (SD) | | Change in steps (SD) | | Change in MVPA (SD) | | Change in SED (SD) | | Change in Steps (SD) | |
|  | β (SD) | P= | β (SD) | P= | β (SD) | P= | β (SD) | P= | β (SD) | P= | β (SD) | P= |
| PM2.5 (SD) | -0.01 (-0.04, 0.03) | 0.770 | **0.05 (0.01, 0.10)** | **0.017*** | 0.00 (-0.04, 0.04) | 0.932 | -0.02 (-0.07, 0.03) | 0.425 | **0.06 (0.01, 0.12)** | **0.026*** | -0.01 (-0.06, 0.04) | 0.754 |
| NO2 (SD) | -0.01 (-0.04, 0.03) | 0.721 | **0.06 (0.02, 0.10)** | **0.006*** | -0.01 (-0.05, 0.03) | 0.574 | -0.05 (-0.11, 0.01) | 0.136 | **0.11 (0.03, 0.18)** | **0.004*** | -0.05 (-0.11, 0.01) | 0.141 |
| PM10 (SD) | -0.01 (-0.05, 0.02) | 0.460 | **0.05 (0.01, 0.10)** | **0.016*** | -0.01 (-0.05, 0.03) | 0.687 | -0.02 (-0.07, 0.02) | 0.323 | **0.06 (0.01, 0.11)** | **0.023*** | -0.01 (-0.05, 0.03) | 0.669 |

*Represents significant values (p<0.05)

Model 1: Standard adjustments (Age, ethnicity, sex, smoking status, past medical history of cardiovascular disease, past medical history of respiratory disease, treatment group, change in wear time between accelerometery measures, SED/MVPA/steps at start of observation, body mass index, season of accelerometer measurements)

Model 2: Standard adjustments, social deprivation, greenspace, measures of built environment (road density, footpath density, junctions, cul-de-sacs, connected intersections), occupational status

**MVPA:** Time spent in moderate-to-vigorous physical activity; **SD:** Standard deviation; **SED:** Time spent in sedentary behaviour; **CI:** Confidence interval; **PM2.5:** Particulate matter ≤ 2.5μm; **NO2:** Nitrogen dioxide; **PM10:** particulate matter ≤ 10.0μm

# Supplementary Table 4: Association between average pollutant concentrations during observation and change in annual change in daily minutes of MVPA/SED/steps

|  | **Model 1** | | | | | | **Model 2** | | | | | | |
| --- | --- | --- | --- | --- | --- | --- | --- | --- | --- | --- | --- | --- | --- |
|  | Change in MVPA (min/day) | | Change in SED (min/day) | | Change in steps (steps/day) | | Change in MVPA (min/day) | | Change in SED (min/day) | | Change in Steps (steps/day) | | |
|  | β (95% CI) | P= | β (95% CI) | P= | β (95% CI) | P= | β (95% CI) | P= | β (95% CI) | P= | β (95% CI) | P= |  |
| PM2.5 (µgm^-3^) | -0.04 (-0.79, 0.72) | 0.926 | **4.77 (1.40, 8.14)** | **0.005*** | -3.75 (-90.46, 82.96) | 0.932 | -0.40 (-1.44, 0.63) | 0.447 | **6.34 (1.62, 11.06)** | **0.008*** | -41.44 (-167.03, 84.15) | 0.518 |  |
| NO2 (µgm^-3^) | -0.03 (-0.16, 0.11) | 0.725 | **0.89 (0.27, 1.50)** | **0.005*** | -3.90 (-19.58, 11.78) | 0.626 | -0.17 (-0.39, 0.05) | 0.130 | **1.43 (0.40, 2.46)** | **0.006*** | -19.40 (-47.25, 8.44) | 0.172 |  |
| PM10 (µgm^-3^) | -0.10 (-0.62, 0.41) | 0.692 | **2.96 (0.62, 5.30)** | **0.013*** | -10.74 (-72.11, 50.63) | 0.732 | -0.22 (-0.82, 0.37) | 0.464 | **3.17 (0.54, 5.81)** | **0.018*** | -22.99 (-93.03, 47.04) | 0.520 |  |

*Represents significant values (p<0.05)

β represents the change in MVPA/SED/steps per 1 µgm^-3^ increasing in pollutant concentration

Model 1: Standard adjustments (Age, ethnicity, sex, smoking status, past medical history of cardiovascular disease, past medical history of respiratory disease, treatment group, change in wear time between accelerometery measures, SED/MVPA/steps at start of observation, body mass index, season of accelerometer measurements)

Model 2: Standard adjustments, social deprivation, greenspace, measures of built environment (road density, footpath density, junctions, cul-de-sacs, connected intersections), occupational status

**MVPA:** Time spent in moderate-to-vigorous physical activity; **SED:** Time spent in sedentary behaviour; **CI:** Confidence interval; **PM2.5:** Particulate matter ≤ 2.5μm; **NO2:** Nitrogen dioxide; **PM10:** particulate matter ≤ 10.0μm

# Supplementary Table 5: Complete case analysis for the associations of exposure to air pollution and annual change in daily minutes of MVPA/SED/steps.

|  | **Model 1** | | | | | |
| --- | --- | --- | --- | --- | --- | --- |
|  | Change in MVPA (min/day) | | Change in SED (min/day) | | Change in steps (steps/day) | |
|  | β (95% CI) | P= | β (95% CI) | P= | β (95% CI) | P= |
| PM2.5 (µgm^-3^) | -0.27 (-1.29, 0.75) | 0.598 | **6.13 (1.38, 10.89)** | **0.011*** | -0.97 (-121.83, 119.89) | 0.987 |
| NO2 (µgm^-3^) | -0.05 (-0.19, 0.10) | 0.530 | **0.95 (0.29, 1.60)** | **0.005*** | -4.17 (-19.92, 11.57) | 0.603 |
| PM10 (µgm^-3^) | -0.34 (-1.11, 0.42) | 0.376 | **4.63 (0.97, 8.30)** | **0.013*** | -13.26 (-104.48, 77.96) | 0.776 |

*Represents significant values (p<0.05)

β represents the change in MVPA/SED/steps per 1 µgm^-3^ increasing in pollutant concentration

Model 1: Standard adjustments (Age, ethnicity, sex, smoking status, past medical history of cardiovascular disease, past medical history of respiratory disease, treatment group, change in wear time between accelerometery measures, SED/MVPA/steps at start of observation, body mass index, season of accelerometer measurements)

**MVPA:** Time spent in moderate-to-vigorous physical activity; **SED:** Time spent in sedentary behaviour; **CI:** Confidence interval; **PM2.5:** Particulate matter ≤ 2.5μm; **NO2:** Nitrogen dioxide; **PM10:** particulate matter ≤ 10.0μm
